# Icariin Promotes the Osteogenesis of Bone Marrow Mesenchymal Stem Cells through Regulating Sclerostin and Activating the Wnt/*β*-Catenin Signaling Pathway

**DOI:** 10.1155/2021/6666836

**Published:** 2021-01-22

**Authors:** Jianliang Gao, Shouyu Xiang, Xiao Wei, Ram Ishwar Yadav, Menghu Han, Weihao Zheng, Lili Zhao, Yichuan Shi, Yanming Cao

**Affiliations:** Department of Orthopedics, The Second Affiliated Hospital of Guangzhou Medical University, Guangzhou, China

## Abstract

Osteoporosis (OP) is a metabolic disease characterized by decreased bone mass and increased risk of fragility fractures, which significantly reduces the quality of life. Stem cell-based therapies, especially using bone marrow mesenchymal stem cells (BMSCs), are a promising strategy for treating OP. Nevertheless, the survival and differentiation rates of the transplanted BMSCs are low, which limits their therapeutic efficiency. Icariin (ICA) is a traditional Chinese medicine formulation that is prescribed for tonifying the kidneys. It also promotes the proliferation and osteogenic differentiation of BMSCs, although the specific mechanism remains unclear. Based on our previous research, we hypothesized that ICA promotes bone formation via the sclerostin/Wnt/*β*-catenin signaling pathway. We isolated rat BMSCs and transfected them with sclerostin gene (*SOST*) overexpressing or knockdown constructs and assessed osteogenic induction in the presence or absence of ICA. Sclerostin significantly inhibited BMSC proliferation and osteogenic differentiation, whereas the presence of ICA not only increased the number of viable BMSCs but also enhanced ALP activity and formation of calcium nodules during osteogenic induction. In addition, the osteogenic genes including Runx2, *β*-catenin, and c-myc as well as antioxidant factors (Prdx1, Cata, and Nqo1) were downregulated by sclerostin and restored by ICA treatment. Mechanistically, ICA exerted these effects by activating the Wnt/*β*-catenin pathway. In conclusion, ICA can promote the proliferation and osteogenic differentiation of BMSCs in situ and therefore may enhance the therapeutic efficiency of BMSC transplantation in OP.

## 1. Introduction

Osteoporosis (OP) is a common skeletal disease characterized by reduced bone mineral density (BMD), bone microstructure deterioration, and an increased risk of fragility fractures [1, 2]. The incidence of OP has risen sharply with the aging of the global population, and currently, one-third of women and one-fifth of men are afflicted worldwide [3]. The antiosteoporosis drugs approved at present are bone resorption inhibitors [4], and their long-term intake can increase the risk of jaw osteonecrosis and atypical femoral fractures, thereby limiting their clinical application [5]. Therefore, it is necessary to develop novel treatment strategies for OP.

Recent studies show that bone marrow mesenchymal stem cells (BMSCs) can repair bone defects in several animal models [6] and are a promising tool for bone regeneration in OP [7]. However, the survival and differentiation rates of the transplanted BMSCs are low, which significantly reduces the efficacy of BMSC-based regenerative therapy [8]. Therefore, enhancing the proliferation and osteogenesis differentiation of BMSCs in situ can significantly improve therapeutic efficacy. Sclerostin is an osteoinhibitory protein encoded by the *SOST* gene that is secreted by bone cells [9] and inhibits osteoblast activity by inactivating the Wnt/*β*-catenin pathway [10]. The *SOST-/-* mice show accelerated osteoblast differentiation via *β*-catenin activation [11], which suggests that knocking down the *SOST* gene may enhance osteogenic differentiation of BMSCs and promote bone renewal.

**Figure 1:**
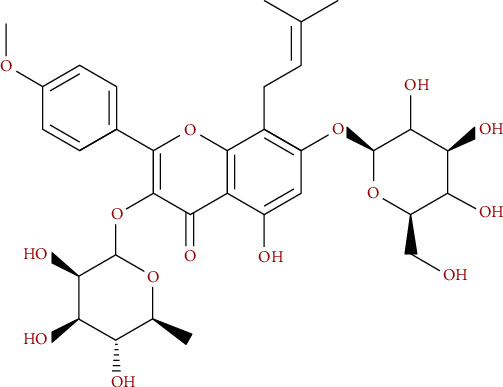
The chemical structure of ICA.

Icariin (ICA, chemical structure shown in Figure 1) is the main active flavonoid glycoside in the Chinese herbal medicine *Herba Epimedii*, which is used to treat renal disorders and prevent bone loss [12]. Recent studies show that ICA can effectively treat postmenopausal OP and promote bone formation [13] by stimulating BMSC proliferation and osteogenic differentiation [14]. In addition, the Chinese medicinal formulation Zuogui pills can also regulate BMSC osteogenic differentiation via the Wnt/*β*-catenin signaling pathway [15]. The aim of this study was to elucidate the mechanistic basis of the proosteogenic function of ICA, with specific focus on the sclerostin-Wnt/*β*-catenin signaling pathway. To this end, we isolated rat BMSCs and transfected them with *SOST* overexpression or knockdown constructs and treated them with ICA during osteogenic induction.

## 2. Materials and Methods

### 2.1. Animals and Ethics

Twelve-week-old female Sprague–Dawley (SD) rats weighing 200–300 g were purchased from the Experimental Animal Center of Sun Yat-sen University (Guangzhou, China, Certificate SCXK (Guangdong) 2016-0029). Animal experiments were performed in accordance with the guidelines of the Regulations of the People's Republic of China on the Administration of Experimental Animals and approved by the Animal Ethics Committee of the Second Hospital Affiliated with Guangzhou Medical University.

### 2.2. Chemical Reagents and Drugs

Dulbecco minimum essential medium (DMEM), fetal bovine serum (FBS), potassium phosphate buffer saline (PBS), and penicillin-streptomycin were purchased from Hyclone (Logan, Utah, USA). Icariin (ICA, purity > 94%) and dimethyl sulfoxide (DMSO) were obtained from Sigma (Steinheim, Germany). The alkaline phosphatase (ALP) staining kit (BCIP/NBT kit) was obtained from Beyotime Biotechnology (Shanghai, China) and the ALP activity measurement kit from Nanjing Jiancheng Biotech (Nanjing, China). Anti-*β*-catenin antibody was purchased from GeneTex (Southern California, USA) and the antibodies targeting GSK-*β* and p-GSK-*β* from GST (Hangzhou, China). Secondary antibodies were obtained from Abcam (Shanghai, China).

### 2.3. BMSC Extraction and Culture

The animals were euthanized by cervical dislocation, and the tibia and femurs were removed. The bone marrow cavities were flushed repeatedly with 10% FBS-supplemented DMEM using a sterile 10 ml syringe to prepare a single cell suspension. The cells were seeded into a 25 cm^2^ flask and cultured at 37°C under 5% CO_2_ [16]. The medium was changed every 3 days, and the cells were observed regularly under an inverted microscope. After culturing for 7–10 days, the 80%–90% confluent monolayer was harvested with 0.25% EDTA-trypsin, washed at 1000 rpm for 5 minutes, and resuspended in complete DMEM for subculturing at 1 : 2 ratio.

### 2.4. Flow Cytometry

BMSCs (1 × 10^6^ cells/ml) were incubated with phycoerythrin-labeled anti-CD90, anti-CD44, anti-CD31, or anti-CD34 antibodies at room temperature in the dark. After washing twice with PBS at 800 rpm for 5 minutes, the cells were acquired by flow cytometry [17].

### 2.5. Vector Construction and Lentivirus Production

The *SOST* overexpression and shRNA lentiviruses were constructed by Suzhou Genewiz. The *SOST* gene was amplified using the following primers: SOST forward ATGCAGCTCTCACTAGCCCCTTGCC and SOST reverse CTAGTAGGCGTTCTCCAGCTCCGCCTGG. The SOST shRNA sequences were as follows: forward GGCCTCCTCAGGAACTAGAGAATTCAAGAGATTCTCTAGTTCCTGAGGAGGCTTTTT and reverse AAAAAGCCTCCTCAGGAACTAGAGAATCTCTTGAATTCTCTAGTTCCTGAGGAGGCC. The sequences were cloned into L303-CMV.Gene.EF1.GFP-T2A-Puro and L202-CMV.CopGFP.2A.Puro H1 plasmids, which were transiently transfected into 293T packaging cells (Invitrogen, Thermo Fisher Scientific Inc.). The serum-free medium was replaced after 8 hours with complete medium. The cells were cultured for 48 hours, and the supernatant was collected and concentrated. The virus titer was measured, and the transduction rate was measured by PCR [18].

### 2.6. BMSC Transfection and Osteogenic Induction

The BMSCs were transduced with the *SOST* overexpression or shRNA lentiviruses and cultured in osteogenic induction medium with or without 0.1 *μ*M ICA. Nontransfected and normal controls were also included. After predetermined time points, osteogenic differentiation was assessed by specific tests.

### 2.7. CCK-8 Assay

BMSCs were seeded into 96-well plates at the density of 1 × 10^4^ per well, and 10 *μ*l CCK-8 reagent (Keki Bio, Cat. No. KGA317) was added to each well after 24, 48, and 72 h of culture. After incubating for 2 hours, the absorbance was measured at 450 nm using a microplate reader (Thermo Fisher Scientific, Vantaa, Finland).

### 2.8. Alkaline Phosphatase (ALP) Staining and Activity Assay

The suitable cells were fixed with 10% formaldehyde for 15 minutes, rinsed with distilled water for 30 seconds, and stained with NBT-BCIP solution (Beyotime Biotechnology, China) at 37°C in the dark for 15 minutes [19]. After washing with distilled water for 30 seconds, the stained cells were viewed under a microscope. For the ALP activity assay, the cells were digested with 0.25% pancrease at room temperature for 2 to 3 minutes and centrifuged. After washing once with PBS, the lysate was analyzed using the ALP assay kit according to the manufacturer's instructions.

### 2.9. Alizarin Red Staining

The differentiated BMSCs were transferred to a six-well plate and fixed with 2 ml 4% neutral formaldehyde solution for 30 min. After rinsing twice with PBS, 1 ml Alizarin red solution was added to each well, and the cells were stained for 3–5 min. The cells were rinsed 2–3 times and observed under a microscope.

### 2.10. RT-qPCR

Total RNA was extracted using Trizol reagent (Invitrogen, USA) and quantified using a Thermo Scientific Microplate Reader (Thermo, USA). RT-qPCR was conducted using SYBR Green qPCR SuperMix (Invitrogen, California, USA) on the ABI PRISM® 7500 Sequence Detection System (Applied Biosystems; Thermo Fisher Scientific, Inc.). The primer sequences are listed in Table 1.

### 2.11. Western Blotting

Western blotting was performed according to standard protocols [20]. Briefly, the proteins were loaded onto 10% SDS-PA gels, electrophoresed, and transferred onto polyvinylidene difluoride membranes (Millipore, USA). The blots were probed overnight with anti-*β*-catenin, anti-GSK-3*β*, and anti-p-GSK-3*β* primary antibodies at 4°C, followed by secondary antibody (1 : 2000 Southern Biotech) at room temperature for 1 hour. GAPDH (1 : 1000, Aksomics, China) was used as the internal control [21]. The positive bands were visualized using an Enhanced Chemiluminescence Western Blot Substrate Kit (Keygen, Nanjing) and analyzed using ImageJ software (National Institutes of Health, Bethesda, MD).

### 2.12. Statistical Analysis

Data are presented as mean ± standard deviation (SD). Statistical analyses were performed using SPSS version 16.0 software (SPSS Inc., Chicago, IL, USA). One-way analysis of variance (ANOVA) or Student's *t*-test was used to compare different groups. *P* values less than 0.05 were considered statistically significant.

## 3. Results

### 3.1. Characterization of BMSCs

**Figure 2:**
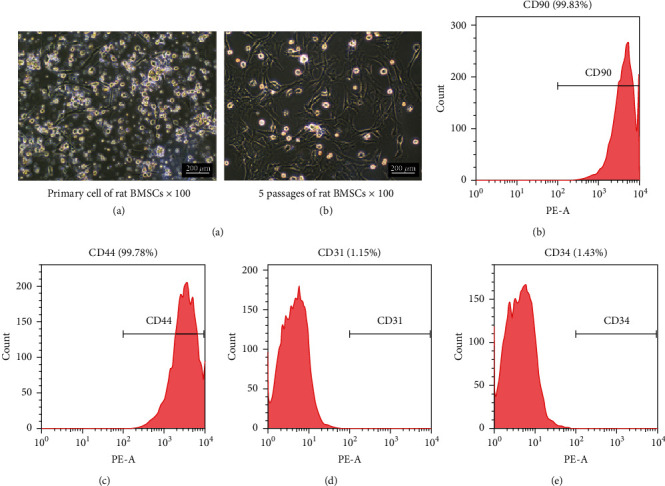
Morphology and phenotypic characterization of BMSCs. (a) Representative images of cultured rBMSCs at the primary passage (A) and passage 5 (B). Flow cytometry plots showing percentage of cells expressing (b) CD90, (c) CD44, (d) CD31, and (e) CD34.

The initial BMSC cultures exhibited adherent cells, colonies, and floating cells (Figure 2(a), A), and the latter disappeared completely by the fifth passage (Figure 2(a), B). Immunophenotypic analysis showed that 99.83% and 99.78% of the cells expressed CD90 and CD44, and only 1.15% and 1.43% expressed CD31 and CD34, respectively (Figures 2(b)–2(e)), indicating that most cells were BMSCs.

### 3.2. ICA Promotes Proliferation and Osteogenic Differentiation of BMSCs

**Figure 3:**
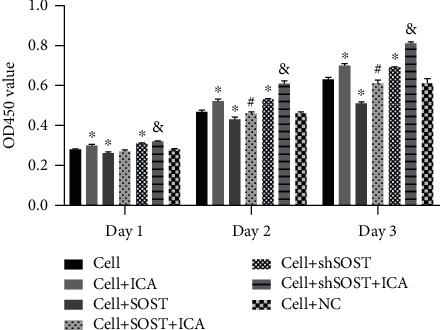
Effects of ICA and sclerostin on BMSC proliferation. Percentage of viable cells in the indicated groups at days 1, 2, and 3 postinduction. All data are presented as mean ± SEM. ^∗^*P* < 0.05 compared to the control group, ^#^*P* < 0.05 compared to the sclerostin overexpression group, and ^&^*P* < 0.05 compared to the sclerostin knockdown group.

**Figure 4:**
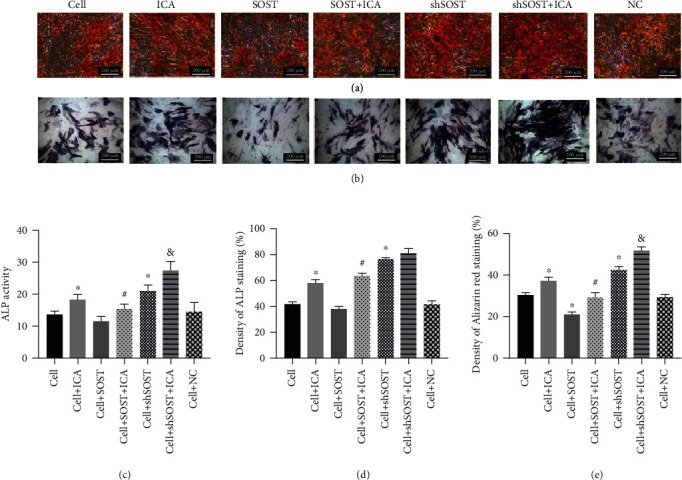
Effects of ICA and sclerostin on the osteogenic differentiation of BMSCs. (a) Alizarin red staining, (b) alkaline phosphatase staining, (c) ALP activity in the indicated groups, and (d, e) ratio of control in ALP staining and Alizarin red staining. All data are presented as mean ± SEM. ^∗^*P* < 0.05 compared to the control group, ^#^*P* < 0.05 compared to the sclerostin overexpression group, and ^&^*P* < 0.05 compared to the sclerostin knockdown group.

As shown in Figure 3, 0.1 *μ*M ICA significantly increased proliferation of the BMSCs compared to the untreated controls. Osteogenic differentiation of the BMSCs was assessed by ALP staining and activity and Alizarin red staining. ICA significantly enhanced ALP levels and activity in the BMSCs compared to the control (Figure 4), which is indicative of early osteoblast differentiation. Furthermore, intense Alizarin red staining in the ICA-treated cells indicated formation of calcified nodules and osteogenic differentiation (Figure 4). Taken together, ICA significantly promoted BMSC proliferation and osteogenic differentiation *in vitro*.

### 3.3. ICA Reverses the Inhibitory Effect of Sclerostin on Osteogenic Gene Expression

**Figure 5:**
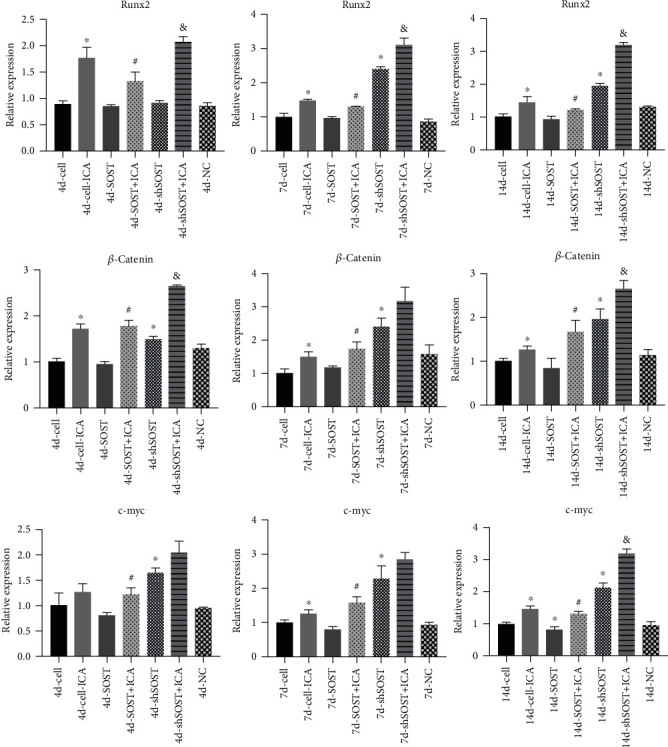
The expression of Runx2, *β*-catenin, and c-myc mRNAs at the different time points (4, 7, and 14 days) of osteogenic induction in the indicated groups. All data are presented as mean ± SEM. ^∗^*P* < 0.05 compared to the control group, ^#^*P* < 0.05 compared to the sclerostin overexpression group, and ^&^*P* < 0.05 compared to the sclerostin knockdown group.

To ascertain the involvement of sclerostin in ICA-induced osteogenic differentiation of BMSCs, we transfected the cells with *SOST* overexpression and shRNA constructs prior to ICA treatment. As shown in Figures 3 and 4, *SOST* overexpression inhibited BMSC proliferation and ALP activity compared to the control group, whereas *SOST* knockdown had the opposite effect. However, ICA treatment restored proliferative and osteogenic capacity of the *SOST*-overexpressing BMSCs, which underscores the proosteogenic activity of ICA. Consistent with this, ICA significantly increased the expression levels of osteogenic genes including Runx2, *β*-catenin, and c-myc after 4, 7, and 14 days. Similar results were observed in the *SOST*-knockdown BMSCs as well. In addition, the osteogenic genes were also upregulated in the *SOST*-overexpressing and *SOST*-knockdown cells following ICA treatment (Figure 5). Taken together, sclerostin inhibits the osteogenic potential of BMSCs, which can be reversed by ICA.

### 3.4. ICA Enhances the Antioxidant Response in BMSCs

**Figure 6:**
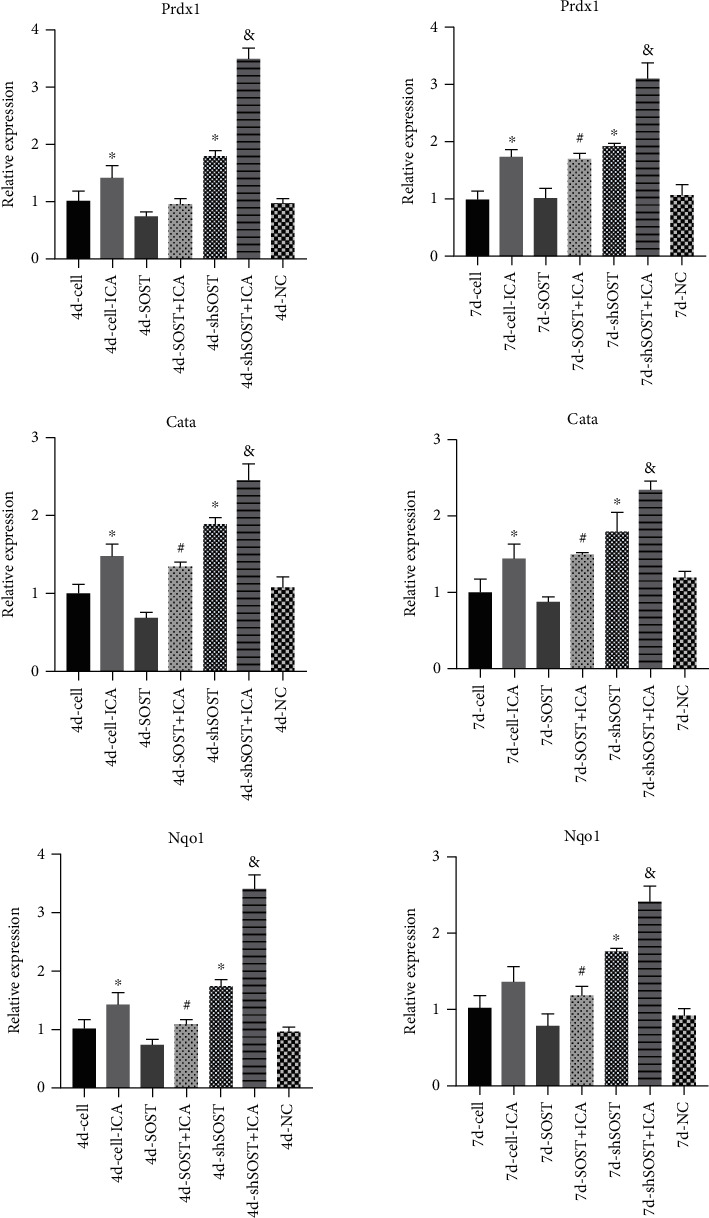
Prdx1, Cata, and Nqo1 mRNA levels in the BMSCs at the different time points (4 and 7 days) of osteogenesis in the indicated groups. All data are presented as mean ± SEM. ^∗^*P* < 0.05 compared to the control group, ^#^*P* < 0.05 compared to the sclerostin overexpression group, and ^&^*P* < 0.05 compared to the sclerostin knockdown group.

Oxidative stress is one of the underlying causes of OP and is known to inhibit osteogenesis [22]. To determine whether ICA modulates oxidative stress in the BMSCs, we analyzed the expression levels of antioxidant factors including Prdx1, Cata, and Nqo1 in the differentially treated cells. As shown in Figure 6, both ICA treatment and *SOST* knockdown significantly upregulated *Prdx1*, *Cata*, and *Nqo1* mRNA levels on days 4 and 7 postosteogenic induction compared to the control group, whereas *SOST* overexpression had the opposite effect. Furthermore, ICA treatment augmented the antioxidant response in the *SOST*-knockdown BMSCs and restored the same in cells overexpressing *SOST*. Taken together, ICA promotes the osteogenic differentiation of BMSCs by mitigating oxidative stress.

### 3.5. ICA Promotes Osteogenesis via the Sclerostin/Wnt/*β*-Catenin Signaling Pathway

**Figure 7:**
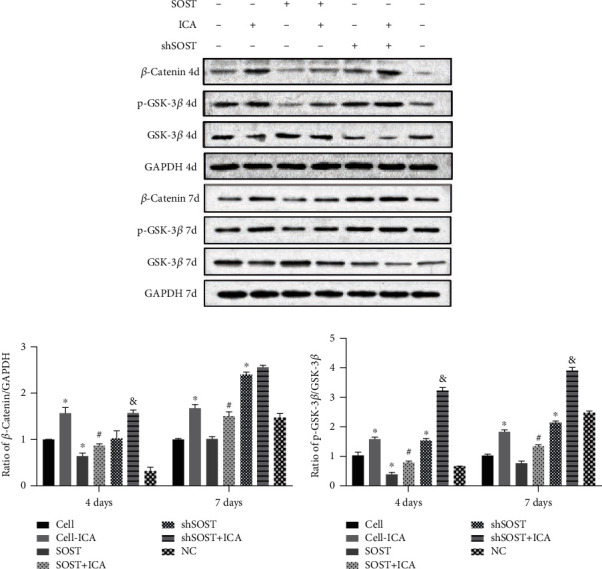
Immunoblot showing *β*-catenin, GSK-3*β*, and p-GSK-3*β* protein levels in the BMSCs cultured for 4 and 7 days. All data are presented as mean ± SEM. ^∗^*P* < 0.05 compared to the control group, ^#^*P* < 0.05 compared to the sclerostin overexpression group, and ^&^*P* < 0.05 compared to the sclerostin knockdown group.

To further elucidate the mechanistic basis of ICA action, we next analyzed the expression levels of key Wnt/*β*-catenin pathway intermediates, such as *β*-catenin, GSK-3*β*, and p-GSK-3*β*. As shown in the immunoblots in Figure 7, both ICA and *SOST*-shRNA significantly upregulated *β*-catenin and p-GSK-3*β* proteins on days 4 and 7 of culture compared to the control group. In contrast, *SOST* overexpression downregulated these factors at the same time points. Consistent with the findings so far, ICA increased the expression of Wnt/*β*-catenin pathway factors in BMSCs regardless of the *SOST* expression status. Taken together, ICA reverses the antiosteogenic activity of sclerostin by activating the Wnt/*β*-catenin signaling pathway.

## 4. Discussion

The incidence of osteoporotic fractures is steadily increasing due to a globally aging population and is associated with reduced quality of life as well as significant socioeconomic burden [23]. Therefore, it is vital to explore novel treatment options for OP that are highly effective and have minimal side effects. We found that sclerostin inhibited BMSC proliferation and osteogenic differentiation, which was reversed by ICA treatment. ICA not only upregulated osteogenesis-related genes including Runx2, *β*-catenin, and c-myc but also increased the expression of antioxidant factors (Prdx1, Cata, and Nqo1). Furthermore, ICA activated the Wnt/*β*-catenin pathway in *SOST*-overexpressing BMSCs. Thus, ICA may promote osteogenic differentiation of BMSCs by activating the sclerostin/Wnt/*β*-catenin signaling pathway.

Sclerostin is secreted by bone cells and inhibits differentiation of osteoblasts [24]. *SOST-/*- mice display accelerated bone formation, as well as increased bone mass and strength [25]. Furthermore, blocking sclerostin with specific antibodies can restore bone mass during estrogen deficiency [26]. To the best of our knowledge, this is the first study to directly assess the role of sclerostin in the osteogenic differentiation of BMSCs *in vitro*. Sclerostin overexpression significantly inhibited the proliferation of rBMSCs and downregulated osteogenic factors, which was neutralized by ICA, indicating a novel regulatory pathway of osteogenic differentiation.

The transcription factor Runx2 is necessary for osteoblast differentiation and bone formation and is an early indicator of the same [27]. Previous studies have shown that Runx2 induces differentiation and migration of osteoblasts and chondrocytes via the PI3K-Akt signaling pathway [28]. c-myc is a key downstream component of the Wnt/*β*-catenin pathway and mediates cell proliferation. Indo et al. [29] reported that the inhibition of c-myc in mature osteoclasts reduces bone-resorbing activity and downregulates the neutral amino acid transporter (B0), which in turn suppresses osteoclastogenesis. We found that sclerostin significantly downregulated Runx2 and c-myc, whereas ICA restored the expression of both. Thus, ICA promotes osteogenic differentiation of BMSCs by regulating the expression of sclerostin.

Oxidative stress plays an important role in osteoporosis [30] by accelerating apoptosis of BMSCs, osteoblasts, and osteoclasts as well as promoting the proliferation and differentiation of osteoclasts [31]. Prdx1, Cata, and Nqo1 are antioxidants [32] that clear ROS and suppress oxidative stress [33]. In the present study, sclerostin significantly downregulated the above factors and ICA had the opposite effect, indicating that inhibition of oxidative stress is one of the mechanisms through which ICA may promote osteogenic differentiation of BMSCs.

**Figure 8:**
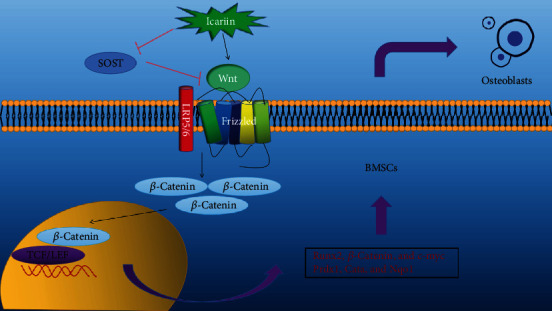
Schematic diagram.

The Wnt/*β*-catenin signaling pathway mediates BMSC proliferation and osteoblast differentiation. Following interaction of the Wnt glycoprotein with frizzled (FZD) and low-density lipoprotein receptor-related protein 5/6 (LRP5/6) [34], axin is recruited to the receptor complex and binds to the phosphorylation site of LRP. This leads to complex dissociation and phosphorylation of glycogen synthase kinase 3*β* (p-GSK3*β*), resulting in the cytosolic accumulation of *β*-catenin and nuclear translocation, eventually leading to the transcriptional activation of the target genes [35]. Sclerostin downregulated the expression of *β*-catenin and p-GSK-3*β*, which were increased by ICA treatment. Thus, ICA promotes osteogenesis via the sclerostin/Wnt/*β*-catenin signaling pathway (schematic diagram shown in Figure 8).

## 5. Conclusions

In summary, ICA promotes osteogenic differentiation of BMSCs by inhibiting the action of sclerostin by activating the Wnt/*β*-catenin pathway. It can potentially enhance the survival and differentiation of transplanted BMSCs in situ and improve the efficacy of BMSC-based regenerative therapy for OP.

## Figures and Tables

**Table 1:** Primers used for target amplification in this study.

Name	Accession number	Primer	Sequence (5′-3′)
Runx2	NM_053470.1	Forward Reverse	GATGCCTTAGTGCCCAAATGT GGCTGAAGGGTGAAGAAAGC
*β*-catenin	NM_053357.2	Forward Reverse	TGAGAAACTTGTCCGATGCA CACTTGGCACACCATCATCT
c-myc	NM_012603.2	Forward Reverse	TGTAGTAATTCCAGCGAGAG CGCAGATTGTAAGTTCCAG
Prdx1	NM_057114.1	Forward Reverse	GGATTGGGACCCATGAACAT GAACTGGAAGGCCTGGACTA
Cata	NM_012520.2	Forward Reverse	CCATCGCCAGTGGCAATTAC AGTCCTTGTGAGGCCAAACC
Nqo1	NM_017000.3	Forward Reverse	TGTGGCTTCCAGGTCTTAGA TGACTCCTCCCAGACAGTCT
GAPDH	NM_017008.3	Forward Reverse	CCCATTCTTCCACCTTTGAT CAACTGAGGGCCTCTCTCTT

## Data Availability

The data used to support the results of this study can be obtained from the corresponding author according to the requirements.
